# Book Review: Enacting Musical Time: The Bodily Experience of New Music

**DOI:** 10.3389/fpsyg.2021.727768

**Published:** 2021-08-09

**Authors:** José L. Besada

**Affiliations:** Department of Musicology, Universidad Complutense de Madrid, Madrid, Spain

**Keywords:** time, enaction, embodied cognition, affordances, listening, contemporary music

The Oxford Studies in Music Theory are recently strengthening the focus on issues around rhythm and musical time (Yust, 2018; Ohriner, 2019). Kozak's volume in this collection pursues this path by scrutinizing contemporary music through the lenses of phenomenology and cognitive science. His principal goal is to reconsider the widespread approach to time by music theorists as external to the listener's situated experience. As stated in the introduction, the author regards musical time as “constituted by the moving bodies of participants engaged in musical activities,” which leads to his main thesis: “musical time emerges when the listener enacts his or her implicit *kinesthetic knowledge* about ‘how music goes”' (p. 4–5).

Chapter 1 criticizes the impact of Newtonian and Cartesian conceptions in music theory for an objective time which spread from the eighteenth century. Kozak embraces in turn the idea of a lived time “as part of the unfolding dynamical system that emerges between an embodied consciousness and the world” (p. 34), thus endorsing the phenomenological tradition by Edmund Husserl and Maurice Merleau-Ponty. Informed by James J. Gibson's and Shaun Gallagher's approaches, Kozak considers the significance of music “in and through the dynamical system that forms when acoustical phenomena elicit responses from enculturated listeners that make these phenomena musical”; consequently, time “is not a condition of music, but something that emerges from it” (p. 53). Affordances are the topic of Chapter 2. Instead of limiting himself to preexistent musicological uses of this term in performance studies, he widens the ecological context by borrowing the notion of social affordances and proposing the temporal ones which jointly specify aesthetic behavior and frame musical affordances. After reviewing Anthony Chemero's approach to radical embodied cognitive science and dynamical systems theory, Kozak specifically defines temporal affordances as “information specified in perceived events in the dynamical relationship between two or more physical affordances,” and highlights their relevance as “temporal alignment […] critical to the successful realization of an intended action” (p. 90). Chapter 3 is mainly devoted to bodily matters. The fundamental contribution in this section is the definition of kinesthetic knowledge as “a contextual enactment of the dynamics, affectivity, and intercorporeality of our bodily involvement with the world,” wherein the body “enact[s] its agency in response to both physical and cultural constraints” (p. 129). I highlight in this chapter his ecological distinction between synchronization and coordination, which is chiefly illustrated with a musical example by Brian Ferneyhough. This distinction leads to privilege the contextual joint action instead of any underlying metrical beat or rhythmic patterns. Merleau-Ponty emerges again in Chapter 4 through the notion of flesh–*la chair –*for depicting the body secreting time via enaction. Particularly, the proposal offered by Kozak moves beyond Merleau-Ponty's predilection of haptic and visual examples toward a phenomenological framework that highlights the experience of listening. Some reasonings in this section are aimed at revealing that, beyond highly rationalized conceptions of musical analysis, “the body is already doing analytical work on its own terms” (p. 183), which speaks to the central role it plays during the enactive listening experience. Chapters 5 and 6 are finally governed by two main case studies–from Louis Andriessen's and Toshio Hosokawa's oeuvre–for further developments of the previous framework. This choice makes these two last chapters quite more meaningful for scholars in the field of music theory than those dealing first and foremost with the psychology of music.

Kozak's argumentative style is clearly rooted into the theoretical production of Zbikowski (2002, 2017), who was his Ph.D. advisor. This is particularly noticeable in the choice of apparently simple musical examples which raise important questions that are addressed through a rigorous methodological framework and with very subtle terminological precision. In addition, in Chapter 3 incorporates some empirical evidence–from research carried out by himself–for supporting some of his reasonings. This direct participation in empirical research is relatively unusual from the side of music theorists. Finally, I consider that a short conclusion by the end would have enhanced the global scope of the whole essay.

Kozak's focus on embodiment and enaction targets the listener's experience of time, which is a very appealing approach for both the music theory and the psychology of music communities. His insistence on embodiment and enaction pushes forward new directions beyond some canonic perspectives (London, 2004; Toussaint, 2013) which have conceptualized time more abstractly. By taking this path, some high-level visual representations of time, like ubiquitous timelines, have been underestimated or overtly neglected, in my opinion, through several pages of his book. However, these kinds of representations are often significant from the composers' side, in a quest of anchoring their particular struggle with temporal conceptions. This topic currently elicits scholarly discussion from cognitive perspectives which acknowledge the importance of embodiment and enaction–from a less radical viewpoint, though–as substantial features of compositional practices (Besada and Pagán Cánovas, 2020; Besada et al., 2021). Rather than a critique of Kozak's position, my last remark is a challenge for future collaboration around overlapping research questions.

## Author Contributions

The author confirms being the sole contributor of this work and has approved it for publication.

## Conflict of Interest

The author declares that the research was conducted in the absence of any commercial or financial relationships that could be construed as a potential conflict of interest.

## Publisher's Note

All claims expressed in this article are solely those of the authors and do not necessarily represent those of their affiliated organizations, or those of the publisher, the editors and the reviewers. Any product that may be evaluated in this article, or claim that may be made by its manufacturer, is not guaranteed or endorsed by the publisher.
